# Local anesthesia versus saddle block for open hemorrhoidectomy: cost-analysis from a randomized, double blind controlled trial

**DOI:** 10.1186/s12913-023-10290-4

**Published:** 2023-11-22

**Authors:** Franck Katembo Sikakulya, Robinson Ssebuufu, Xaviour Francis Okedi, Moris Baluku, Herman Lule, Sonye Magugu Kiyaka, Joshua Muhumuza, Selamo Fabrice Molen, Godefroy Nyenke Bassara, Musa Abbas Waziri, Stephen Mbae Kithinji, Mugisho Munyerenkana Leocadie, Byamungu Pahari Kagenderezo, Jeannot Baanitse Munihire, Bienfait Mumbere Vahwere, Ahmed Kiswezi, Patrick Kyamanywa

**Affiliations:** 1 Faculty of Clinical Medicine and Dentistry, Department of Surgery, Kampala International University Western Campus, Ishaka-Bushenyi, Uganda; 2 Faculty of Medicine, Université Catholique du Graben, Democratic Republic of the Congo, Butembo, Democratic Republic of Congo; 3 Department of Surgery, Mengo Hospital, Kampala, Uganda; 4 Department of Anesthesia and critical care, Kabale University, Kabale, Uganda; 5 Department of Surgery, Kiryandongo Hospital, Kiryandongo, Uganda; 6 Department of Clinical Neurosciences, University of Turku, Turku, Finland; 7 State Specialist Hospital Maiduguri, Maiduguri, Borno State Nigeria; 8 Uganda Martyrs University, Nkozi, Uganda

**Keywords:** Cost analysis, Operative time, 3rd or 4th degree hemorrhoids, open hemorrhoidectomy, Local anesthesia, Saddle block, Uganda

## Abstract

**Background:**

Despite the benefits attributed to the use of local anesthesia (LA) for open hemorrhoidectomy (OH) in developed countries, this technique is still not considered as the first line technique in low-income countries such as Uganda; therefore, we aimed at comparing the cost of OH under LA versus Saddle block among patients with 3rd or 4th degree hemorrhoids.

**Methods:**

This trial was conducted from December 2021 to May 2022 among patients with primary uncomplicated 3rd or 4th degree hemorrhoids. The operating time, and direct costs in (US$) including medical and non-medical were recorded. We analysed the cost in the two groups (local anesthesia versus saddle block) using SPSS version 23.0.

**Results:**

Findings of fifty-eight patients were analysed including 29 participants per group. There was a significant difference in operating time and cost among the two groups (p < 0.05). The mean operating time was 15.52 ± 5.34(SD) minutes versus 33.72 ± 11.54 min for OH under LA and SB respectively. The mean cost of OH under LA was 57.42 ± 8.90 US$ compared to 63.38 ± 12.77US$ in SB group.

**Conclusion:**

The use of local anesthesia for OH was found to have less operating time with high-cost effectiveness. Being affordable, local anesthesia can help to increase the turnover of patients who would otherwise wait for the availability of anesthesia provider. Policy makers should emphasize its applicability in low-income settings to help in the achievement of 2030 global surgery goals.

**Trial registration:**

Pan African Clinical Trials Registry, PACTR202110667430356. Registered on 08/10/2021.

## Background

Open hemorrhoidectomy (OH) (Milligan and Morgan technique) has remained the standard of hemorrhoid surgery worldwide [1]. Hemorrhoidectomy is a common surgical procedure, often associated with significant postoperative pain, and a remarkable economic burden [2, 3]. Open hemorrhoidectomy under local anesthesia (LA) has been shown to have lower complication rates and more cost effective by saving anesthetics for other surgeries compared to saddle block (SB). In addition, OH under LA has been found to increase patient turn over because of the shorter operative time [4, 5].

The major problem in performing hemorrhoidectomy under LA is the pain that occurs during injection of the local anesthetic through the sensitive anoderm [6, 7]. On the other hand, general anesthesia (GA) or SB are associated with complications, requires preoperative preparation and postoperative hospitalization for observation till full recovery [4, 5].

The costs of anesthetic procedures have become an important factor in the selection of the most appropriate technique for anal surgeries [2]. Saddle block requires a trained anesthetic provider to be used for open hemorrhoidectomy and leads to long hospital stays [5, 8] which increases the cost related to OH compared to the use of local anesthesia for the same procedure [2, 5, 6, 9].

The choice of anesthetic technique depends on its lower complication rates, cost effectiveness, and not impinging on active productivity in terms of patient turnover, hence the need to compare the two techniques against these factors. Numerous financial costs can be avoided by day care surgery; for instance, reduction of admission costs, limited use of ward-based facilities plus decreased medication costs [3]. Despite the benefits associated to the use of LA for OH in developed countries, this technique is still not considered as first line in low-income countries such as Uganda, despite the country’s resource constraints. Studies are needed to highlight the importance of local anesthesia for open hemorrhoidectomy in resource limited setting. Therefore, this study aimed at comparing the cost of OH under LA versus SB among patients with 3rd or 4th degree hemorrhoids in three major hospitals in rural Western Uganda.

## Methods

This cost benefit analysis is part of a randomized controlled trial that aimed at comparing outcome of open hemorrhoidectomy using local anesthesia versus saddle block among patients with primary uncomplicated 3rd or 4th degree hemorrhoids in Western Uganda; thus, part of the methodology has been previously described [10] and will be referred to appropriately in the present study.

### Study design

This study was an economic evaluation by cost-benefit analysis from a double-blind randomized controlled trial, conducted in the departments of surgery at Kampala International University-Teaching Hospital (KIU-TH), Kitagata and Adventist Hospital in Western Uganda. The study was approved by the KIU local ethics committee (KIU-REC-2021-24) and registered with *Pan African Clinical Trials Registry (PACTR202110667430356*).

### Study population and sample size determination

Fifty-eight patients with uncomplicated 3rd or 4th degree hemorrhoids were randomized from 1st October 2021 to 2nd June 2022 to undergo OH either under local anesthesia (group A) or the saddle block (group B); (29 patients per group). Detailed methodology including patient selection, sample size determination and analyses have been reported in previous studies [10, 11]. We considered cost analysis of OH as a secondary outcome from this trial [11]. We calculated the cost benefit analysis of OH in both groups based on the different selected hospital surgical tariffs due to lack of a standardized health cost measurement reference in the country. The in-hospital direct and indirect costs for all patients who underwent OH in the two groups alongside with the operating time and length of stay were prospectively documented.

### Cost calculation

During this trial, we considered direct costs, including both medical and non-medical for all patients who underwent OH in the two groups. The materials used during and after open hemorrhoidectomy in both groups such as surgical blades, gloves, drugs among others, and the anesthetic fees were considered as medical direct costs. The non-medical direct costs were those not directly accountable to patients such as: administration, hospital stay, nursing care, files, among others. A total of 58 envelopes were obtained, half of the envelopes contained a chit with letter A for LA and a chit with letter B for saddle block. All financial data are expressed in US dollars (1 US$ = 3729.50 Ugandan shillings) (updated on 22nd June 2022).

### Data analysis

Data was statistically analysed using IBM Statistics SPSS for Windows 23.0. Quantitative data on direct, indirect, and total costs were presented as mean with standard deviation (SD). The mean operative time and standard deviation were computed for each technique of open hemorrhoidectomy. The difference in means was compared using the independent samples t-test and its corresponding two-tailed p-value, regarding p < 0.05 as statistically significant. A bottom-up table of mean charges by group was made to get the mean of cost in the two groups (A and B). A cross tabulation was performed between the two open hemorrhoidectomy techniques to allow for categorical cost-effective analysis. The difference in means was compared using the t-test and its corresponding two-tailed p-value, regarding p < 0.05 as statistically significant.

## Results

### Overview of the findings

**Fig. 1:**
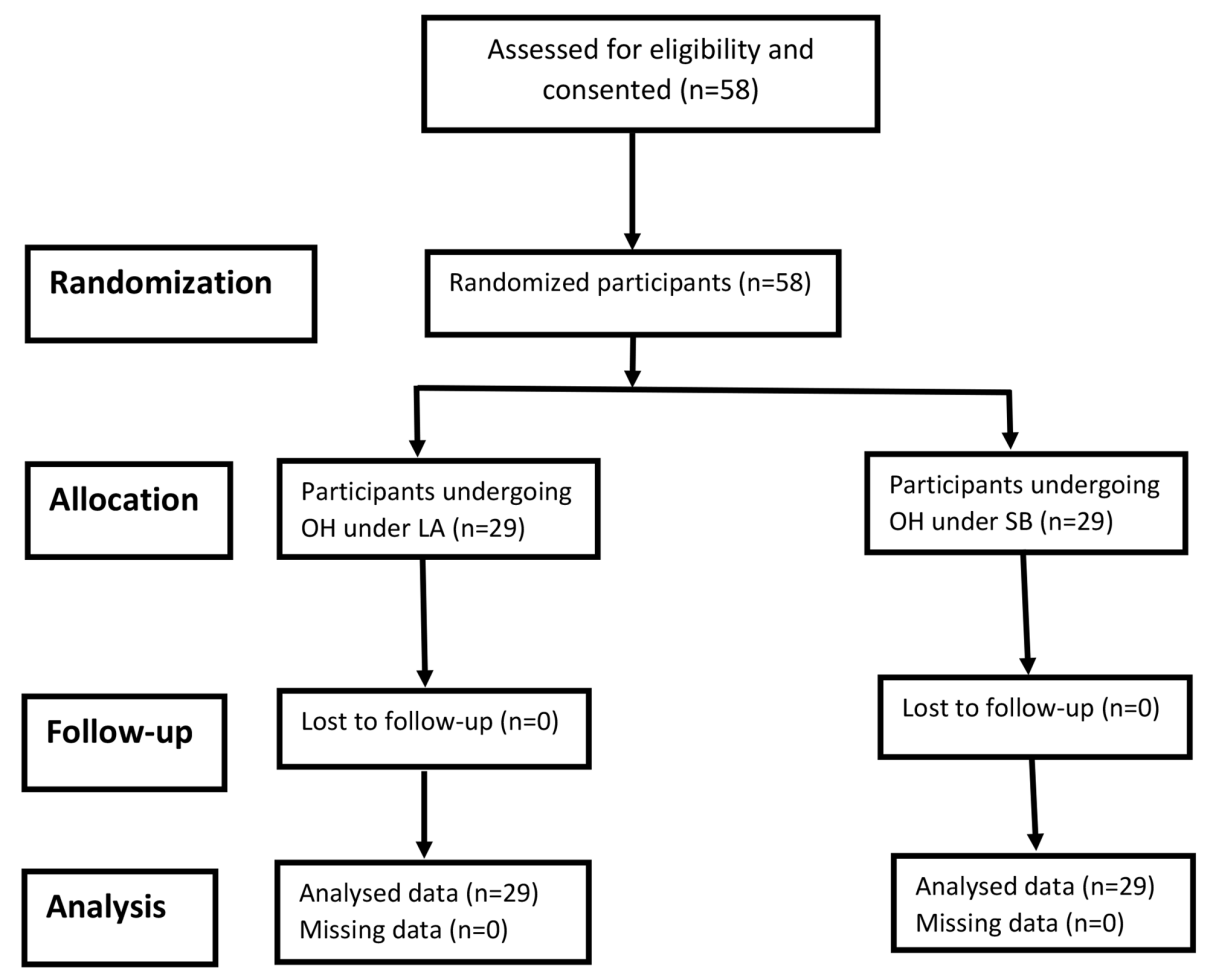
Consolidated standards of reporting trials flow diagram

The Consolidated Standards of Reporting Trials (CONSORT) diagram for patients’ recruitment is shown in the figure below. All participants (58) were randomized and followed up to day 7 post OH. The cost analysis was done without any missing data (Fig. 1).

### Operating time and cost analysis for open hemorrhoidectomy

Of the 58 patients included in this trial, 29 participants per group of anesthesia were considered. The operating time and hospital stay were significantly shorter in group A compared to group B (p < 0.001). The cost of OH was significantly lower in group A compared to group B (p = 0.04) (Table 1).

**Table 1:** Operating time and cost analysis among patients undergoing open hemorrhoidectomy in the two groups

Variables	Group A (n = 29)	Group B (n = 29)	t	P-value
**Operating time (in min)**			-7.713	< 0.001*
Minimum-maximum	10–33	20–74		
Mean ± SD	15.52 ± 5.34	33.72 ± 11.54		
**Hospital Stay (in hours)**			-7.419	< 0.001*
Minimum-maximum	10–40	12–72		
Mean ± SD	20.86 ± 6.46	40.14 ± 12.41		

*P value < 0.05; t: Independent sample t test; SD: standard deviation

### Bottom-up charge technique for OH under LA versus OH under SB

The Table  (2) below shows the charges by group of patients who underwent OH in both groups. The results are presented in terms of mean with standard deviation per variable category.

**Table 2:** Mean cost related to open hemorrhoidectomy by bottom-up charge technique per patient per group

Variable	Group A (Mean ± SD)	Group B (Mean ± SD)	Mean difference	P value
**Surgical related cost** (Surgical blades, sutures, etc.)	14.35 ± 2.22	15.84 ± 3.19	1.49	0.040
**Anesthetic related cost** (Anesthesia fee, anesthetic drugs, and spine needle)	22.97 ± 3.56	25.35 ± 5.11	2.38	0.042
**Medicine related cost** (Antibiotics, analgesics, and fluids)	8.61 ± 1.34	9.51 ± 1.92	0.89	0.045
**Surgical Sundries related cost** (Gloves, syringes, canulation, urinary catheter, etc.)	4.59 ± 0.71	5.07 ± 1.02	0.48	0.044
**Hospital related cost** (Hospital stay cost, nursing care, file, etc.)	6.89 ± 1.07	7.61 ± 1.53	0.72	0.043
**Overall Mean Cost Per Patient**	57.42 ± 8.90	63.38 ± 12.77	5.96	0.044

## Discussion

In our cost-analysis comparison of OH under LA versus SB among patients with 3rd or 4th degree hemorrhoids at three major hospitals in rural Western Uganda, we found that the operating time was greater for SB compared to LA. This is contrary to findings of Younes et al. in Egypt [6] and to those of Sharma et al., [12] where there was no significant difference in mean operating time between the use of LA versus SB for OH [6]. This trial confirms that SB increases duration of operative time for open hemorrhoidectomy compared to local anesthesia in agreement with previous studies [8, 13].

The use of local anesthesia has been shown to be impactful in terms of reducing the cost of surgery though day care surgical approach across the world [14]. This cost-savings though shorter hospital stays and early return to comfortable home environment is critical for resource constrained countries with limited hospital admission bed capacity [14].

In our analysis, we found that the use of LA was significantly associated with low overall cost of OH compared to SB. Our findings are comparable to studies in England and Bangladesh respectively, in regards to shorter hospital stay and costs related to the use of LA versus SB for OH [5, 9], confirming that numerous charges could be avoided by opting for LA in well selected patients. Open hemorrhoidectomy in most low- and middle-income countries (LMICs) is performed under saddle block which requires a trained anesthetic provider in the face of scarce human resources for health. Moreover, saddle block is associated with delay in the initiation of surgery by maintaining the patient in sitting position for an average of five to ten minutes, amidst its other concerns such as: postoperative urinary retention, neural injury, direct nerve and spinal cord injury, cauda equina syndrome, epidural hematoma, post-dural puncture headache, failed block, and epidural abscess [5, 9, 15]. These complications increase hospital length of stay and morbidity [9].

The results of the present trial are also supported by Shaw & Ternent who documented that use of LA was associated with lower financial burden compared to other types of anesthesia for day care surgery by reducing the admission costs, minimal use of ward-based facilities plus decreased medication charges [3]. In addition, it is known that local anesthesia for day care surgery can save up to 25 to 50% of surgical cost compared to other techniques which necessitate long in-hospital stay [3, 16]. This evidence was further supported by a recent meta-analysis [17]. However, our recent study established that LA was associated with slightly higher pain threshold reported by patients following open hemorrhoidectomy for primary uncomplicated 3rd or 4th degree hemorrhoids at 2 h (visual analogue scale 2.28 ± 1.3 LA vs. 1.69 ± 0.09 SB, p = 0.05) [10]. Although this difference was marginal, could imply additional cost implications for LA such as due to stronger analgesics that might be required in the immediate post-operative period. Despite this hiccup, the possibility of switching from LA to SB intra-operatively should ideally make LA more feasible as the first anesthesia option for uncomplicated hemorrhoids [18].

The policy implication of these findings is that the low cost of OH under LA if adopted could protect against impoverishment from direct out-of-pocket payments for surgical and anesthesia care in LMICS. Further, this could increase the surgical volume to fulfill a minimum of 5000 procedures per 100 000 population by 2030 as per the goal of global surgery 2030 [19]. However, despite potential advantages of safety, access, and cost; there are several implementation issues to consider before fully endorsing LA. First, shifting from SB to LA needs to be ground rooted right from the time of training for both medical and anesthesia students and junior professionals which has implications for curriculum development, standardizing, and harmonizing the choice of anesthesia protocols across public and private facilities in LMICs. Secondly recruitment and retention of such trained surgical and anesthesia providers as well as drug stock-outs have been earlier identified as a main obstacle to using local and regional anesthesia according to a study that evaluated barriers to regional anesthesia in LMICs [20]. Lastly, there is need to address poor adaptive leadership to tackle patient, physician, and institutional-related barriers to use of LA. Patient education regarding making autonomous safer anesthesia choices, up-skilling health workers to develop competence in LA and regional blocks and overcoming institutional preferences over individual patient and physicians’ autonomy in procurement and management of anesthesia and surgical materials have been identified as key areas to enhance safe anesthesia and surgery in a broader context [21].

### Study strengths and limitations

This trial had some limitations but also considerable strengths. In terms of limitations, the use of bottom-up technique for cost analysis of the two techniques was the major limitation in this study and this was due to lack of a standardized locally available price reference tool in the country for open haemorrhoidectomy under local anaesthesia versus saddle block. However, despite the above limitations, this study provides evidence-based data for the average cost of OH in rural setting of Uganda, which is one of the developing countries. Noteworthy, the findings from this trial are largely generalizable to hospitals in LMICs with similar settings.

## Conclusion

This trial established that the operating time as well as direct and indirect costs were statistically lower among patients undergoing OH using LA compared to OH under SB. The government in collaboration with the non-governmental organisations should update the guidelines on management of uncomplicated third and fourth-degree hemorrhoids in low-income countries such as Uganda to embrace the use of local anesthesia since it is safe and affordable compared to saddle block.

## Data Availability

The datasets used and/or analysed during the current study is available from the corresponding author on reasonable request.
